# Ensemble neural network modelling for stratified HbA1c prediction: Integrating past glucose measurement as a predictor of glycaemic control

**DOI:** 10.6026/973206300213941

**Published:** 2025-10-31

**Authors:** Prakruti Dash, Kasala Farzia, Dharashree Priyadarshini, Saurav Nayak

**Affiliations:** 1 Department of Biochemistry, All India Institute of Medical Sciences, Bhubaneswar, Odisha, India; 2 Department of Biochemistry, IMS & SUM Hospital Campus-II, Phulnakhara, Bhubaneswar, Odisha, India

**Keywords:** HbA1c prediction, fasting blood glucose, post-prandial blood glucose, ensemble neural network, multi-layer perceptron, glycaemic control, machine learning, diagnostic accuracy

## Abstract

Accurate assessment of glycemic control is crucial for effective diabetes management and the prevention of long-term complications. This study employed an ensemble neural network framework, combining a Multi-Layer Perceptron Regressor (MLPR) and Classifier (MLPC) model, to predict and stratify HbA1c using routine fasting (FBS) and post-prandial (PPBS) glucose values from retrospective e-laboratory data (n = 22,920, 2021-2024). The regressor, trained on mean FBS and PPBS values from the preceding three months, achieved an R^2^ of 81 ± 3.7%, sMAPE of 9.13 ± 4.01% and RMSE of 1.1 ± 0.01, reflecting high predictive accuracy and minimal bias. Partial Dependence and ICE analyses revealed a strong, consistent positive association of FBS with HbA1c across glycaemic ranges. The classifier, based on predicted HbA1c, achieved 87.4% accuracy, 94.3% precision and a Diagnostic Odds Ratio of 35.26 ± 0.36, as confirmed by ROC analysis, which demonstrated superior discrimination compared to traditional glucose metrics.

## Background:

Diabetes mellitus is a chronic, progressive disorder marked by abnormalities in glucose metabolism. In recent years, due to global social and economic development and enhanced living standards, the incidence and prevalence of diabetes have escalated annually, emerging as a significant public health concern that has garnered the attention of governments, health authorities and medical professionals worldwide [1]. As the disorder progresses, the majority of T2DM patients will experience varying degrees of complications, significantly diminish their quality of life and impose a substantial economic burden on their families. The degree of problems is intrinsically linked to glycaemic control. Consequently, proactive, secure and efficient regulation of blood glucose is crucial for minimizing problems, enhancing the quality of life for T2DM patients and alleviating the economic burden on both patients and society [2]. Consequently, prepared, reliable and efficacious management of blood glucose is crucial for minimizing problems, enhancing the quality of life for T2DM patients and alleviating the financial burden on both patients and society. Fasting blood sugar (FBS), postprandial blood sugar (PPBS) and glycosylated haemoglobin (HbA1c) are essential metrics for the clinical diagnosis and assessment of therapy efficacy in T2DM [3]. HbA1c is one of the most widely recognized biomarkers for the diagnosis and management of T2DM. Haemoglobin, a protein found in red blood cells, is associated with glucose molecules in the bloodstream. As blood glucose levels rise, a greater amount of haemoglobin will get glycosylated. The HbA1c test quantifies one's mean blood glucose levels over a span of 90 to 120 days, according to the usual lifespan of red blood cells, which is approximately three months. The American Diabetes Association (ADA) categorizes a HbA1c test value of less than 5.7% as non-diabetic, values ranging from 5.7% to 6.4% as pre-diabetic and a HbA1c value of 6.5% or over as diabetic [4]. In contrast to conventional statistical models that depend on established assumptions about data distribution or linear relationships, Machine Learning models may manage non-linear interactions and incorporate a diverse array of factors. This adaptability enables machine learning to encompass the intrinsic variability of type 2 diabetes mellitus, influenced by several circumstances [5]. By revealing concealed patterns in health data, machine learning algorithms offer more tailored and precise forecasts of individual patient responses to certain treatments [6, 7- 8]. Therefore, it is of interest to elucidate the role of separate blood glucose measurement over the past three months in effectively predicting the HbA1c levels and thus aiding monitoring of glycaemic control in patients through ML Neural Network, Machine Learning Perceptron (MLP) model.

## Methodology:

## Study design and setting:

The study was conducted as a retrospective cross-sectional study based on data mined from e-laboratory records of the Department of Biochemistry at AIIMS Bhubaneswar from the period 2021 to 2024. The Institutional Ethical Body of AIIMS Bhubaneswar provided the ethical clearance. Each HbA1c record was considered a datapoint and the records were tracked backwards to find which datapoints had corresponding glucose measured (FBS or PPBS or both) in the ensuing past three months.

## Prediction workflow:

The prediction of glycaemic control occurs in two distinct steps: (a) MLP-Regressor (MLPR) and (b) MLP-Classifier (MLPC). The regressor model utilizes the mean of all FBS and PPBS measurements over the past three months to predict the HbA1c value. The classifier model uses this predicted HbA1c value to classify into the three categories: Controlled (HbA1c < 7%) and Uncontrolled (HbA1c > =7%).

## Dataset:

The overall dataset is split into two sets by a 70-30 split for training and testing. After the Regressor function, the dataset is randomized and re-split for the classification function similarly. The dataset contains HbA1c values, calculated Mean Plasma Glucose (eAG), mean FBS, mean PPBS and Median of Mean Glucose (Median of mean FBS and PPBS).

## MLP models:

In the current pipeline, the MLPR is configured with three hidden layers, each containing 64 neurons and uses the tanh activation function. It employs the Adam optimizer with an adaptive learning rate strategy and includes an L2 regularisation (alpha) of 0.001 to prevent overfitting. The model is set to train for a maximum of 1000 iterations. For classification, the MLPC is used with two hidden layers of 64 neurons each, utilizing the ReLU activation function and the Adam solver. The classifier is configured to run for up to 1000 iterations, with the default regularisation (alpha = 0.0001). Both models use a fixed random state of 7280 to ensure reproducibility of results.

## Evaluation metrics:

The evaluation of the predictive pipeline was conducted in two distinct stages: regression and classification. The regression model, built using a Multi-Layer Perceptron Regressor (MLP Regressor), was assessed using a robust framework incorporating 5-fold stratified cross-validation combined with 1000-times bootstrapping. The performance of the regressor was quantified using several standard error metrics: R^2^ (coefficient of determination) to measure explained variance, MAPE (Mean Absolute Percentage Error) and sMAPE (symmetric MAPE) to capture relative prediction errors, MSE (Mean Squared Error) and RMSE (Root Mean Squared Error) for absolute error magnitude, MAD (Mean Absolute Deviation) for dispersion of prediction errors and Bias to capture systematic under- or overestimation. Subsequently, the classification model, trained on predicted HbA1c values (and also compared across other features), was evaluated using the same cross-validation and bootstrapping framework. The classifier's performance was assessed using a comprehensive suite of metrics. As with the regressor, all classifier metrics were expressed as mean ± SD over the bootstrapped cross-validation runs, providing a stable and statistically meaningful performance summary.

## Results:

The dataset contained 22920 unique datapoints, which were then split into training (n = 16044) and testing (n = 6876) datasets. The parameter characteristics of both datasets have been tabulated in Table 1 (see PDF). The MLPR model was used to generate a predicted HbA1c value by utilizing the previously measured FBS and PPBS results for the person. The model worked quite well as the predicted value showed an R2 of 81 ± 3.7%, with a sMAPE of 9.13 ± 4.01%. The Bias was very minimal (0.033 ± 0.04) with a low RMSE of 1.1 ± 0.01. To visualize the effect of the measurement of mean FBS and PPBS on the ensuing HbA1c prediction, an integrated Partial Dependence Plot (PDP) and Individual Conditional Expectation (ICE) plot were plotted for the classifications based on HbA1c. It has been shown in Figure 1 (see PDF). The PDP and ICE analyses revealed distinct glycaemic response patterns across the three groups. In diabetics, both FBS and PPBS showed a strong positive influence on HbA1c predictions, with notable inter-individual variability, particularly at higher FBS (>180 mg/dL) and PPBS (>300 mg/dL) levels. Pre-diabetics demonstrated a milder but still upward trend, with subtle heterogeneity in HbA1c response starting from intermediate FBS (110-130 mg/dL) and PPBS (120-150 mg/dL) values. In the normal group, both FBS and PPBS showed minimal impact on HbA1c, with tight clustering of ICE curves, reflecting stable glycaemic control. Overall, FBS contributed more consistently to HbA1c across all groups, while PPBS effects were more individualized and prominent only at extreme values, particularly in the diabetic group. Following the regression model, the MLPC classified the population based on HbA1c stratification. The same was modelled by using the predicted HbA1c values produced by MLPR. The classifier model showed very high accuracy (87.4%) and precision (94.3%). The DOR of MLPC was 35.26 ± 0.36, which was higher than that of using mean FBS (25.24 ± 0.40) or mean PPBS (24.20 ± 0.51) individually. This was also shown by the ROC Curve analysis (Figure 2 - see PDF), where the Area Under Curve for MLPR (0.918, p<0.001) fared statistically better than both mean FBS (0.883, p<0.001; compared to MLPR: z = -32.7, ΔAUC = 0.035, p<0.001) and PPBS (0.890, p<0.001; compared to MLPR: z = -31.3, ΔAUC = 0.025, p<0.001) in predicting glycaemic control.

## Discussion:

The Multi-Layer Perceptron Regressor model shows that it can reliably predict HbA1c levels using easily accessible measurements of Fasting Blood Sugar and Postprandial Blood Sugar. The model explains a large part of the variation in HbA1c, as shown by the high R2 value of 81%. This implies that there is a strong link between these glucose measurements and overall glycaemic control. The model's ability to accurately and precisely predict HbA1c levels is further supported by the low sMAPE of 9.13% and RMSE of 1.1. For diabetes management and personalized treatment plans to work, this level of accuracy is very important [3, 9]. The combined Partial Dependence Plot and Individual Conditional Expectation analyses give us useful information about how FBS and PPBS affect HbA1c predictions in different glycaemic groups. The different patterns seen in diabetics, pre-diabetics and people with normal glycaemic control show how these factors interact in complex ways and how important it is to take individual differences into account when making clinical decisions. The Multi-Layer Perceptron Classifier's classification of the population based on predicted HbA1c values shows how well this method works. The Diagnostic Odds Ratio of 35.26 shows that the classifier model is very accurate and precise. This means that the predicted HbA1c values are good signs of how well blood sugar is controlled. Also, the fact that the MLPR did better in the ROC curve analysis than either the mean FBS or PPBS alone shows how valuable it is to include these measurements in a predictive model. These results are in line with other research that shows how machine learning models can use a higher degree of data and find complicated relationships to make HbA1c predictions more accurate [10, 11-12]. The MLPR model has three hidden layers, each with 64 neurons and uses the tanh activation function and L2 regularisation to keep the model from overfitting. This shows how complex architecture needs to be in order to capture these relationships. Using the Adam optimizer with a learning rate that changes over time makes the model work even better [13, 14]. Many studies have looked into using machine learning to predict HbA1c levels. Alhassan *et al.* showed that using machine learning models with Electronic Health Records data to predict the risk of HbA1c elevation was promising [10]. They got an accuracy of 83.22% with a multilayer perceptron model. In the same way, Al-hussein *et al.* created hybrid models that combined logistic regression and random forests to improve the accuracy of HbA1c predictions [5]. They were able to reach 93% accuracy using a small set of key performance indicators like age, BMI, HDL and triglycerides. Alazwari *et al.* did a similar study and found that machine learning models could predict the onset of Type 1 Diabetes (T1D) in children by finding key performance indicators [12]. These studies, along with our reported observations, show that machine learning models could make more accurate and personalized predictions of changes in HbA1c. This could help clinicians plan better treatment charts for people with diabetes and suffering from its complications [10, 11, 15 and 16]. This becomes especially important in our current scenario, because T2DM affects about 537 million adults around the world and that number is expected to rise to 783 million by 2045 [9, 17]. This will pose a serious public health crisis in developing countries that will necessitate the urgent need for accurate predictive methods. Other machine learning methods have also shown promise for predicting diabetes. For example, Albadri *et al.* used a hybrid machine learning algorithm to make a model that predicts diabetes [18]. Sonko *et al.* looked at a number of machine learning methods for predicting diabetes early on and found that neural networks worked very well [14]. Farran *et al.* used non-invasive parameters and machine learning algorithms to figure out how likely someone is to get Type 2 Diabetes in the future [16]. These studies all point to the fact that machine learning can be a useful way to find diabetes early on. Wearable devices and machine learning models have also been able to predict blood glucose levels, which makes it easier to keep track of and treat diabetes [2, 13]. More and more people are realizing that AI methods like machine learning and deep learning could be useful for predicting long-term diabetes risk [14]. However, our study had its limitations. The model's performance is based on a certain set of data and it may not work the same way in other populations or clinical settings without greater thrust on testing and data-cycling in a more heterogeneous multi-centric manner. Also, relying on FBS and PPBS measurements may not show all the factors that affect HbA1c levels. A greater degree of prospective research should look into adding more variables to the predictive model, like lifestyle factors, genetic predispositions and other relevant clinical data. This will make the model more accurate and reliable. In addition, it is important to look into how well the model works in different healthcare settings to see if it can be used in the real world in the field, clinic and community.

## Conclusion:

Ensemble neural network models using routine fasting and post-prandial glucose data can accurately predict and stratify HbA1c, enabling individualized glycemic assessment is shown. The interpretability of MLP models through PDP and ICE analyses highlights their clinical transparency and applicability in real-world settings. This framework shows strong potential for integration into clinical and community workflows, with future expansions to include behavioural and clinical variables and a forthcoming web-based application for broader accessibility.
